# Long-term visual outcomes and development of retinal pigment epithelial and outer retinal atrophy in neovascular age-related macular degeneration: a comparison of conventional and novel classifications

**DOI:** 10.1186/s12886-026-05255-0

**Published:** 2026-08-24

**Authors:** Debabrata Hazra, Yohei Tomita, Atsuro Uchida, Soshi Kato, Norihiro Nagai, Misa Suzuki, Sakiko Minami, Hiromitsu Kunimi, Norimitsu Ban, Toshihide Kurihara, Hajime Shinoda, Yoko Ozawa, Kazuno Negishi

**Affiliations:** 1 Department of Ophthalmology, Keio University School of Medicine, 35 Shinanomachi, Shinjuku-ku, Tokyo, 160-8582 Japan; 2 Department of Ophthalmology, Teikyo University School of Medicine University Hospital, Mizonokuchi, Takatsu-ku, Kawasaki, Japan; 3 Department of Ophthalmology, Federation of National Public Employees Mutual Aid Associations Tachikawa Hospital, Tachikawa, Tokyo Japan; 4 Department of Ophthalmology, Saiseikai Central Hospital, Minato-ku, Tokyo, Japan; 5 Department of Ophthalmology and Visual Science, Yokohama City University School of Medicine, Kanazawa-ku, Yokohama, Japan; 6 Tomioka Eye Clinic, Kanazawa-ku, Yokohama, Japan; 7 Department of Clinical Regenerative Medicine, Fujita Medical Innovation Center Tokyo, Ota-ku, Tokyo, Japan

**Keywords:** Retinal pigment epithelium and outer retinal atrophy (RORA), Complete RORA (cRORA), Neovascular age-related macular degeneration (nAMD), Anti-VEGF therapy

## Abstract

**Background:**

Retinal pigment epithelial and outer retinal atrophy (RORA) and complete RORA (cRORA) are major determinants of long-term vision in neovascular age-related macular degeneration (nAMD), but their development across subtypes is poorly defined, especially in Asian populations, in whom pachychoroid-driven disease is common. We compared long-term visual outcomes and atrophy burden between the conventional and novel nAMD classifications.

**Methods:**

One hundred twenty eyes of 120 treatment-naïve patients with nAMD followed at least six years were classified by conventional (typical nAMD [tAMD], polypoidal choroidal vasculopathy [PCV], retinal angiomatous proliferation [RAP]) and novel (drusen type, pachychoroid type, mixed type, other type) schemes. All eyes received three loading anti-VEGF injections followed by a pro re nata regimen. RORA within the central 1 mm was graded on optical coherence tomography; lesions ≥ 250 μm were defined as cRORA. Visual acuity (VA) retention, atrophy prevalence, and VA around atrophy onset were assessed.

**Results:**

Mean age was 78.0 ± 9.2 years and overall VA retention was 71%. VA retention did not differ among conventional subtypes, but under the novel classification was significantly lower in the drusen type and higher in the pachychoroid type (*P* < 0.01). VA declined significantly after both RORA and cRORA onset (both *P* < 0.001). Final RORA/cRORA prevalence was 44.4%/29.6% in tAMD, 50.0%/28.3% in PCV, 83.3%/50.0% in RAP and 76.9%/61.5% in the drusen type, in which cRORA was highest (*P* < 0.05).

**Conclusions:**

The novel classification distinguished long-term visual outcomes that the conventional classification did not: the drusen type showed the poorest VA retention and highest cRORA prevalence, whereas the pachychoroid type fared best.

## Background

Neovascular age-related macular degeneration (nAMD) is one of the leading causes of visual acuity deterioration among the elderly population globally [1]. It is characterized by choroidal neovascularization (CNV), resulting in rapid and progressive loss of central vision [2]. The advent of anti-vascular endothelial growth factor (anti-VEGF) therapy has revolutionized the treatment landscape for nAMD, significantly improving both visual outcomes and quality of life for affected patients [3]. Despite these advancements, the long-term efficacy and safety of anti-VEGF therapy, as well as the optimal management strategies for different subtypes of nAMD, remain not fully understood.

The conventional classification of nAMD includes three primary subtypes: typical nAMD (tAMD), polypoidal choroidal vasculopathy (PCV), and retinal angiomatous proliferation (RAP) [4]. These subtypes are distinguished based on clinical and angiographic features, such as polypoidal lesions and branching vascular networks in PCV or retinal-retinal and retinal-choroidal anastomoses in RAP [5, 6]. However, this classification only partially captures the disease’s heterogeneity, particularly regarding underlying pathophysiological mechanisms and therapeutic responses.

Recently, the concept of “pachychoroid spectrum disease” has emerged, highlighting a different pathological mechanism compared to the nAMD seen in Caucasian populations, which typically involves soft drusen as precursor lesions [7]. Asian populations have a substantially higher prevalence of PCV and pachychoroid-related neovascular diseases, both of which may have different patterns of retinal atrophy and responses to therapy compared with typical drusen-driven AMD [8]. Yanagi et al. proposed a novel classification system for nAMD that is based on the presence of soft drusen and pachychoroid disease characteristics [9]. This system categorizes nAMD into four groups: drusen-related macular neovascularization (MNV), pachychoroid-related MNV, mixed MNV, and non-drusen non-pachychoroid MNV. Drusen-related MNV is characterized by large, indistinct drusen, while pachychoroid-related MNV involves focal or diffuse choroidal thickening, increased choroidal vessel permeability, and pachydrusen. Mixed MNV includes both drusen-related and pachychoroid-related disease features, while non-drusen non-pachychoroid MNV lacks these specific features.

Although anti-VEGF therapy has been an effective treatment for nAMD, a higher incidence of overall retinal pigment epithelial and outer retinal atrophy (RORA) and complete RORA (cRORA) has been reported in the long run [10]. RORA and cRORA are significant complications associated with AMD, which are characterized by photoreceptor degeneration, retinal pigment epithelium (RPE) loss, and choroidal hypertransmission [11]. Overall RORA denotes any lesion fulfilling CAM criteria and that cRORA denotes the subset with a minimum linear diameter of at least 250 μm. However, this kind of hypertransmission is also observed in macular atrophy (MA) [12], a significant event that may occur in eyes with nAMD treated with anti-VEGF therapy [13]. Here, we regarded the hypertransmission area of MA as RORA and cRORA of nAMD. Our recent study has shown that cRORA involvement in foveal center was related to BCVA deterioration [14]. On the other hand, another study has indicated that nAMD patients treated with anti-VEGF therapy retained good visual acuity retention despite the higher incidence of RORA and cRORA [15]. Other research has found that a higher frequency of injections was associated with a slower progression of atrophy, suggesting that more frequent administration of anti-VEGF therapy may mitigate the advancement of cRORA [16]. As the natural history and risk factors for atrophy in Asian patients with neovascular AMD might not be identical to those observed in Western cohorts [8], evaluating RORA and cRORA in Asian nAMD patients would lead to the discovery of new therapeutic strategies for them.

Here, we investigated the emergence of retinal pigment epithelial and outer retinal atrophy (RORA) and complete RORA (cRORA) in Japanese patients with different subtypes of nAMD undergoing anti-VEGF therapy. This study compared conventional and novel classification systems to test whether the novel classification better captures long-term visual prognosis and atrophy burden, therapeutic outcomes of Asian patients with neovascular AMD. Our results could underscore the importance of individualized treatment plans and the potential benefits of integrating novel classification criteria into clinical practice for improved management of nAMD.

## Methods

### Study Design and Study Populations

This retrospective cohort study adhered to the tenets of the Declaration of Helsinki and was approved by the Keio University School of Medicine Ethics Committee (approval number 20100003). Details of ethical approval and the informed consent procedure are provided in the Declarations section.

The inclusion criteria were treatment-naïve patients with nAMD who received the anti-VEGF treatment (using either pegaptanib, ranibizumab, or aflibercept; protocol: 3 loading doses followed by pro re nata (PRN)) regimen at the Keio University Hospital from January 2012 to December 2015 and completed at least 6 years of follow-up. Exclusion criteria included treatment with photodynamic therapy (PDT) or anti-VEGF treatment before 2012 at any hospital, patients who were not followed up for six years due to referral to another physician, and patients not correctly adhering to the treatment protocol. For patients in whom anti-VEGF treatment was administered to both eyes during the study period, only the first-treated eyes were included to avoid inter-eye correlation in this single-eye-per-patient analysis. For patients who had been followed up for more than six years, only data from the first six years after the start of treatment was used.

### Measurements

We performed fluorescein angiography (FA) and indocyanine green angiography (ICGA). We obtained fundus photography to diagnose nAMD and classify its disease type for every patient before starting the anti-VEGF therapy. They also underwent best-corrected visual acuity (BCVA) measurements at the baseline before anti-VEGF therapy and every subsequent visit during the follow-up period. We also acquired images of spectral-domain optical coherence tomography (SD-OCT) (SPECTRALIS^®^, HRA, Heidelberg INC, Heidelberg, Germany) to evaluate the emergence of RORA and cRORA at the baseline and every subsequent visit. Trained orthoptists performed all examinations. BCVA retention was defined as an increase of less than 0.3 in LogMAR BCVA from baseline to the final visit because this corresponds approximately to a loss of fewer than 15 ETDRS letters (three lines), which has been widely accepted as the definition of vision maintenance in pivotal neovascular AMD trials [1, 17].

### Conventional and Novel Classifications of nAMD

**Fig. 1:**
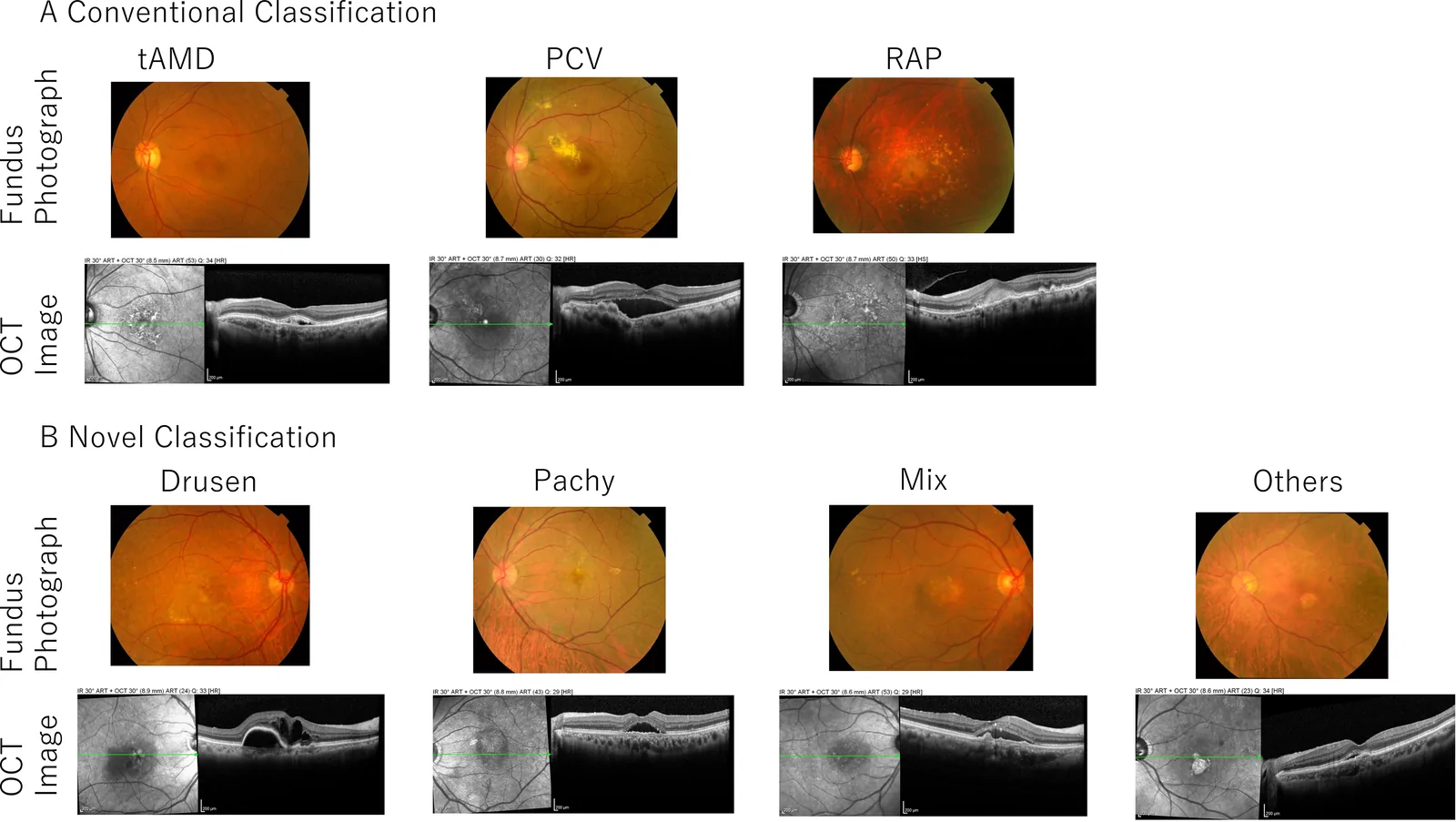
Conventional and Novel Classifications of neovascular age-related macular degeneration (nAMD). **(A)** Representative fundus photographs and optical coherence tomography (OCT) images illustrating the conventional classification of nAMD. For the conventional classification, we classified those that had polypoidal lesions with or without branching vascular networks as polypoidal choroidal vasculopathy (PCV) and that exhibited retinal-retinal or retinal-choroidal anastomoses as retinal angiomatous proliferation (RAP), and that were diagnosed with neither PCV nor RAP were classified as typical nAMD (tAMD). **(B)** Representative fundus photographs and OCT images illustrating the novel classification of nAMD. For the novel classification, we classified the macular neovascularization (MNV) into four groups according to the presence of soft drusen with a diameter of 125 μm or more and indistinct boundaries, and the presence of pachychoroid-related disease characteristics: Drusen-related MNV (Drusen type), Pachychoroid-related MNV (Pachy type), Mixed MNV (Mix type), and non-Drusen non-Pachychoroid MNV (Others type)

From the results of fluorescent fundus angiography and fundus photography images, we classified the patients in two ways: by the conventional classification and the novel classification proposed by Yanagi [9] of nAMD. For the conventional classification, we classified those that had polypoidal lesions with or without branching vascular networks as PCV, those that exhibited retinal-retinal or retinal-choroidal anastomoses as RAP, and those that were diagnosed with neither PCV nor RAP as tAMD [5, 6] (Fig. 1A) For the novel classification, we classified the macular neovascularization (MNV) into four groups according to the presence of soft Drusen with a diameter of 125 μm or more and indistinct boundaries, and the presence of pachychoroid-related disease characteristics: Drusen-related MNV (drusen type), Pachychoroid-related MNV (pachychoroid type), Mixed MNV (mixed type), and non-Drusen non-Pachychoroid MNV (others type) [9, 18] (Fig. 1B) Characteristics of pachychoroid-related disease included focal or diffuse choroidal thickening, increased permeability of choroidal vessels in late ICGA, dilated pachyvessels in Haller’s layer, thinning of the choroidal capillary plate and Sattler’s layer, CSC- and PPE-like retinal pigment epithelial damage [9, 18]. Hard drusen with a diameter of 63 μm or less and well-defined borders were not included in the criteria [9, 18]. Pachydrusen were defined by the following characteristics: (1) diameter of 125 μm or more, (2) irregular outline and well-defined borders, (3) scattered in the posterior pole, (4) yellowish white color and isolated or present as several clusters [19]. These were considered features of pachychoroid-related diseases [9, 18]. Cases with the pachychoroid-related disease characteristics described above, but which also had soft drusen and other characteristics of tAMD, were classified as the mixed type. In contrast, cases without pachychoroid-related disease characteristics and soft drusen were classified as the other type [9, 18]. The drusen type broadly corresponds to a drusen-dominant, typical AMD phenotype; the pachychoroid type overlaps with pachychoroid-driven neovascular disease, including many eyes with PCV or pachychoroid neovasculopathy features; the mixed type includes both soft drusen and pachychoroid-related characteristics, including pachydrusen; and the other category includes non-drusen, non-pachychoroid MNV, into which atypical entities such as some RAP cases may fall.

## Anti-VEGF Therapy Protocol

Twelve retina specialists were involved in the follow-up. The choice and switch of VEGF inhibitor were based on the independent judgment of the treating specialist, but all treatment decisions were made by the treating retina specialists under a shared institutional 3 + PRN regimen during this period [18]. Exudative changes were defined as the presence of new hemorrhages on fundus examination, intraretinal fluid (IRF), subretinal fluid (SRF) on OCT, or an increase in serous subretinal pigment epithelial fluid (sub-RPE fluid) [18].

### Definitions of RORA and cRORA

We defined RORA according to the classification of atrophy meetings (CAM) report 4 that included the following features: (1) hypertransmission of signal into the choroid and (2) a corresponding zone of attenuation or disruption of the RPE, and (3) degeneration of overlying photoreceptor. RORA that extends over 250 μm in diameter was defined as cRORA [11]. We evaluated the presence of RORA and cRORA within a 1 mm diameter from the fovea.

### Statistical Analysis

We calculated the six years’ BCVA retention rate of each type of nAMD under anti-VEGF therapy and compared them between types classified by the conventional classification and the novel classification (chi-square test with subsequent post hoc analysis). Continuous baseline variables, including age and the number of anti-VEGF injections, were compared among subtypes using the Kruskal–Wallis test, because normality of the distributions could not be assumed given the small subgroup sizes. BCVA was compared between the last visit immediately before the first documentation of RORA and cRORA and the visit at which RORA/cRORA was first documented (Wilcoxon signed-rank test). The paired BCVA analysis was restricted to eyes for which BCVA was available both at the visit immediately preceding and at the visit of first documentation of RORA or cRORA; therefore, the number of eyes in this analysis is smaller than the total number of eyes that developed atrophy. Prevalence and new emergence rate of RORA and cRORA during this period were evaluated for each type of nAMD, and we examined their difference among types classified by the conventional classification and the novel classification (chi-square test with subsequent post-hoc analysis). All statistical analyses were performed using statistical analysis software (R version 4.3.0, R Foundation for Statistical Computing, Vienna, Austria). All *P* values less than 0.05 were considered statistically significant. For prevalence and emergence rates, which are proportions, error bars represent the standard error derived from the binomial distribution, calculated as the square root of p(1 − p)/n, where p is the observed proportion and n is the number of eyes at risk in each subtype.

## Results

### Patient Demographics

A total of 120 eyes from 120 consecutive treatment-naïve patients with nAMD were included in the study. The age of the 120 patients (68.3% male) was 78.0 ± 9.2 years (mean ± standard deviation; range, 54–95 years). The mean number of anti-VEGF therapies during the examination period was 16.6 ± 11.3 (1–45). By the conventional classification, the number of patients classified as tAMD type, PCV type, and RAP type were 54 (45.0%), 60 (50.0%), and 6 (5.0%), respectively. By the novel classification, the number of patients classified as pachychoroid type, drusen type, mixed type, and other type were 66 (55.0%), 13 (10.8%), 14 (11.7%), and 27 (22.5%), respectively. Number of patients finally evaluated as having RORA and cRORA were 59 (49.2%) / 36 (30.0%), respectively. The mean age, sex ratio and the number of anti-VEGF therapies of each type of nAMD are shown in Table 1 (conventional classification) and Table 2 (novel classification). No statistically significant differences in age, sex ratio, or number of anti-VEGF therapies were observed between types in either classification.

**Table 1:** Baseline characteristics of study participants (Conventional Classification)

Conventional Classification	All *N* = 120	tAMD *N* = 54	PCV *N* = 60	RAP *N* = 6	*P* value
Age, year (SD)	78.0 (9.2)	77.9 (9.3)	80.0 (9.4)	79.3 (5.6)	> 0.10*
Gender/men, N (%)	82 (68.3)	42 (77.8)	37 (61.7)	3 (50.0)	> 0.10^†^
Number of anti-VEGF therapies (SD)	16.6 (11.3)	15.2 (10.7)	18.2 (12.0)	13.2 (8.7)	> 0.10*

Data are expressed as mean values. tAMD = typical neovascular age-related macular degeneration; PCV = polypoidal choroidal vasculopathy; RAP = retinal angiomatous proliferation; SD = Standard Deviation. Continuous variables were compared among subtypes using the Kruskal–Wallis test (*) or chi-square test (†)

**Table 2:** Baseline characteristics of study participants (Novel Classification)

Novel Classification	All *N* = 120	Pachychoroid *N* = 66	Drusen *N* = 13	Mixed *N* = 14	Other *N* = 27	*P* value
Age, year (SD)	78.0 (9.2)	76.3 (9.4)	82.8 (10.6)	74.3 (9.0)	81.6 (7.4)	> 0.10*
Gender/men, N (%)	82 (68.3)	47 (71.2)	9 (69.2)	6 (42.9)	20 (74.1)	> 0.10^†^
Number of anti-VEGF therapies (SD)	16.6 (11.3)	17.5 (11.9)	18.6 (11.0)	17.7 (12.4)	13.3 (9.2)	> 0.10*

Data are expressed as mean values. SD = Standard Deviation. Continuous variables were compared among subtypes using the Kruskal–Wallis test (*) or chi-square test (†)

### 6-Year BCVA Retention Rate of Different Types of nAMD under Anti-VEGF Therapy

**Fig. 2:**
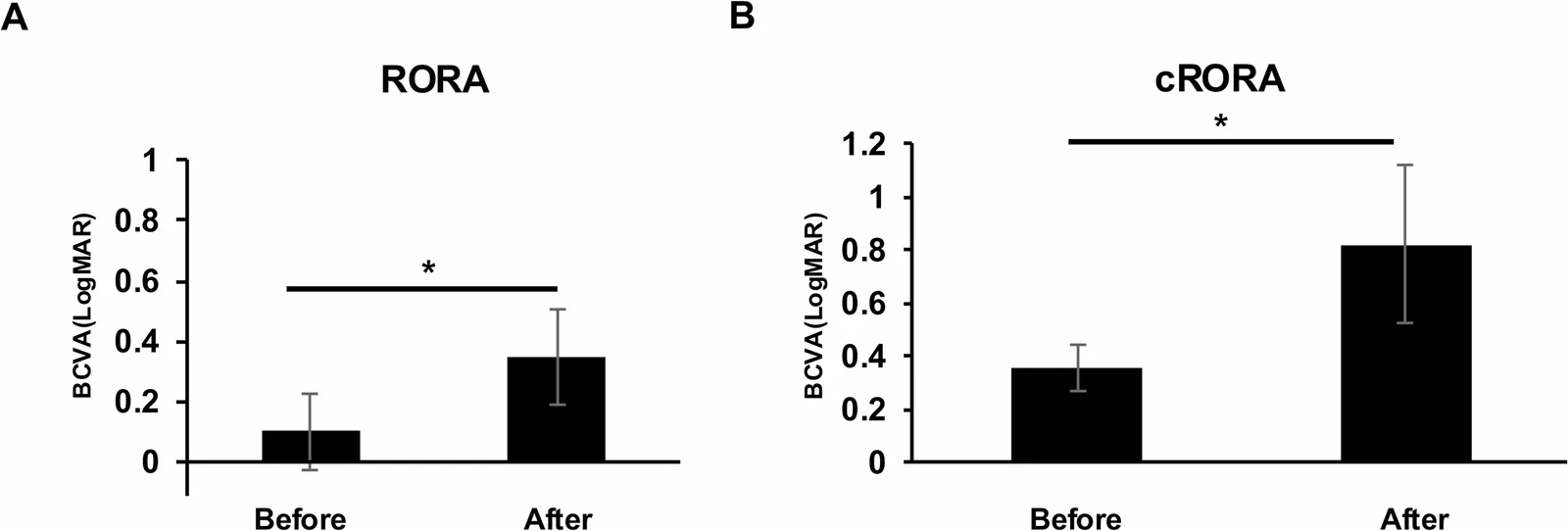
Changes in best-corrected visual acuity (BCVA) before and after the emergence of retinal pigment epithelium and outer retinal atrophy (RORA) and complete RORA (cRORA). **A**) The BCVA values (LogMAR) before and after the emergence of RORA; **B**) the cRORA. The emergence of RORA and cRORA was associated with significant worsening in BCVA values (LogMAR) (RORA: *n* = 26, Wilcoxon signed-rank test, *P* < 0.001; cRORA: *n* = 22, Wilcoxon signed-rank test, *P* < 0.001). Each error bar indicates standard deviation

The mean LogMAR BCVA values at the baseline and the last visit of the examination period were 0.15 ± 0.33 (-0.079-1.3) and 0.35 ± 0.47 (-0.079-2.0), respectively, and the BCVA retention rate was 71%. The LogMAR value of mean BCVA at the baseline, at the last visit of the examination period, and the BCVA retention rate of each type of nAMD were shown (Table 3: the conventional classification; Table 4: the novel classification). Chi-square test with subsequent post-hoc analysis showed no significant difference in BCVA retention rate between types under the conventional classification. However, a significantly lower BCVA retention rate was found in the drusen type and a significantly higher rate in the pachychoroid type compared to other types under the novel classification (tAMD: *n* = 54, PCV: *n* = 60, RAP: *n* = 6, *P* > 0.1; drusen: *n* = 13, pachychoroid: *n* = 66, mix: *n* = 14, others: *n* = 27, *P* < 0.01). We also compared the BCVA between the last visit immediately before the first documentation of RORA and cRORA and the visit at which RORA/cRORA was first documented for all examined patients. We found a significant increase in BCVA values (LogMAR) from 0.10 ± 0.13 (-0.079-0.40) to 0.35 ± 0.16 (-0.079-0.70) and from 0.36 ± 0.08 (-0.079-0.52) to 0.82 ± 0.30 (0.52–1.7) by the RORA and cRORA emergence, respectively (RORA: *n* = 26, Wilcoxon signed-rank test *P* < 0.001; cRORA: *n* = 22, Wilcoxon signed-rank test *P* < 0.001) (Fig. 2).

**Table 3:** Mean BCVA at baseline and last visit (Conventional Classification)

Conventional Classification	All *N* = 120	tAMD *N* = 54	PCV *N* = 60	RAP *N* = 6	*P* value
BCVA, LogMAR (SD)					
Baseline	0.15 (0.33)	0.13 (0.29)	0.17 (0.35)	0.19 (0.50)	> 0.10 ^*^
Last visit	0.35 (0.47)	0.29 (0.43)	0.35 (0.46)	0.76 (0.70)	> 0.10^*^
Change	0.19 (0.36)	0.16 (0.33)	0.19 (0.35)	0.57 (0.62)	
Maintained vision at last visit, N (%)	85 (71)	39 (72)	44 (73)	2 (33)	> 0.10^†^

Data are expressed as the mean. tAMD = typical neovascular age-related macular degeneration; PCV = polypoidal choroidal vasculopathy; RAP = retinal-choroidal anastomoses as retinal angiomatous proliferation; SD = Standard Deviation. Values were compared by Kruskal-Wallis analysis (*) or chi-square test (^†^). Maintained vision at last visit was defined as an increase of less than 0.3 in LogMAR BCVA

**Table 4:** Mean BCVA at baseline and last visit (Novel Classification)

Novel Classification	All *N* = 120	Pachychoroid *N* = 66	Drusen *N* = 13	Mixed *N* = 14	Other *N* = 27	*P* value
BCVA, LogMAR (SD)						
Baseline	0.15 (0.33)	0.16 (0.36)	0.31 (0.43)	0.004 (0.14)	0.12 (0.25)	> 0.10 ^*^
Last visit	0.35 (0.47)	0.32 (0.45)	0.72 (0.59)	0.18 (0.17)	0.32 (0.47)	> 0.10^*^
Change	0.19 (0.36)	0.15 (0.32)	0.41 (0.61)	0.18 (0.25)	0.19 (0.35)	
Maintained vision at last visit, N (%)	85 (71)	54 (82)	5 (38)	9 (64)	17 (63)	< 0.01^†^

Data are expressed as the mean. SD = standard deviation. Values were compared by Kruskal-Wallis analysis (*) or chi-square test (^†^). Maintained vision at last visit was defined as an increase of less than 0.3 in LogMAR BCVA. *P* value less than statistically significant level (< 0.05) is marked in bold

### Changes of RORA and cRORA Prevalence for Different Types of nAMD under Anti-VEGF Therapy

**Fig. 3:**
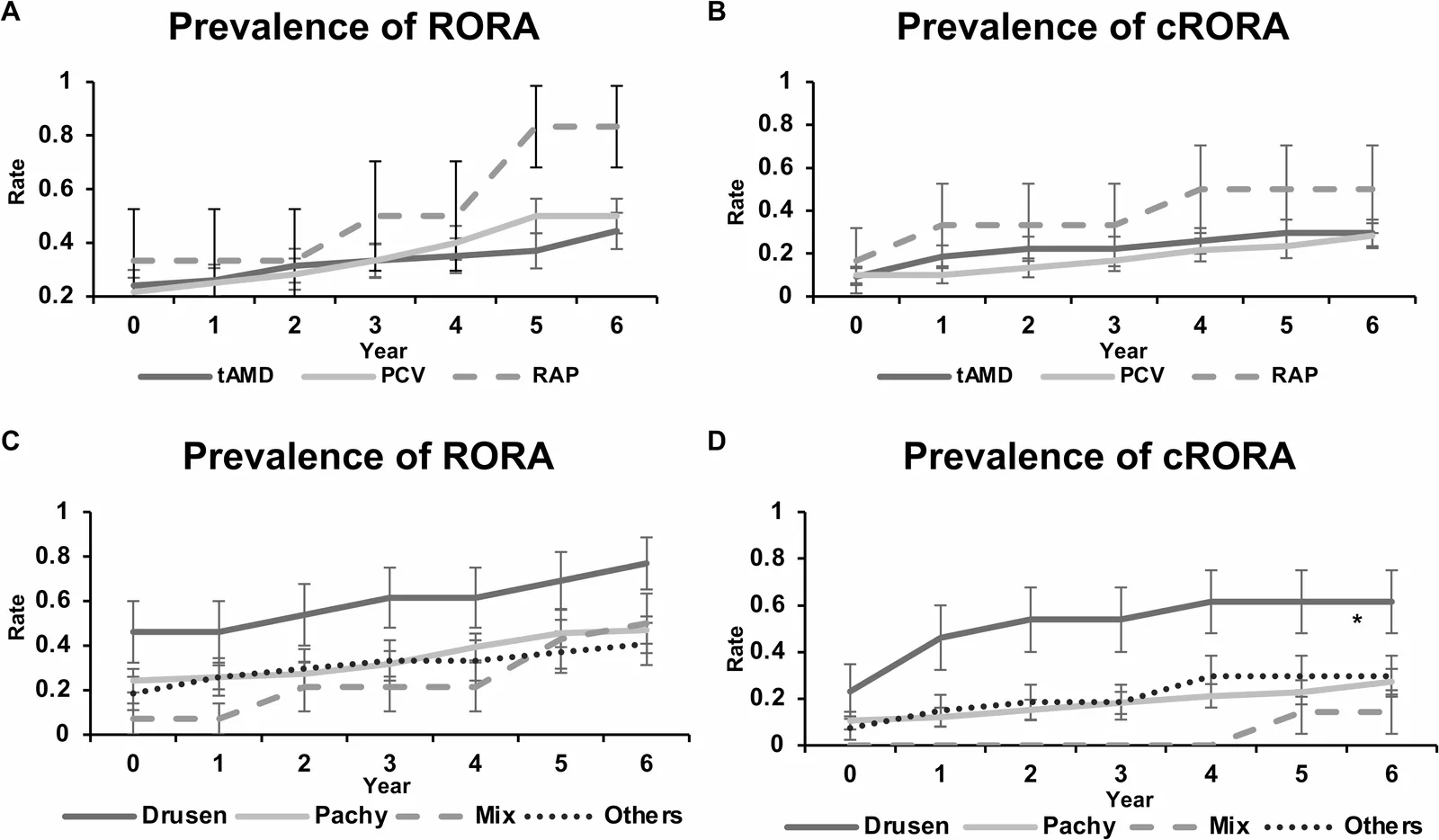
Changes in the prevalence for retinal pigment epithelium and outer retinal atrophy (RORA) and complete RORA (cRORA) in different types of neovascular age-related macular degeneration (nAMD) under anti-vascular endothelial growth factor (anti-VEGF) therapy. **(A)** Prevalence changes in RORA for nAMD patients classified by the conventional classification; **(B)** Prevalence changes in cRORA. The chi-square test showed no significant difference in both RORA and cRORA prevalence between types (RORA: typical AMD (tAMD): *n* = 54, polypoidal choroidal vasculopathy (PCV): *n* = 60, retinal angiomatous proliferation (RAP): *n* = 6, *P* > 0.05; cRORA: tAMD: *n* = 54, PCV: *n* = 60, RAP: *n* = 6, *P* > 0.05). **(C)** Prevalence changes in RORA for nAMD patients classified by the novel classification; **(D)** Prevalence changes in cRORA. The chi-square test with subsequent post-hoc analysis showed a significantly higher rate of cRORA prevalence at the last year of the examination in the drusen type compared with other types but no significant differences were observed in RORA prevalence between types (RORA: drusen: *n* = 13, pachychoroid: *n* = 66, mix: *n* = 14, others: *n* = 27, *P* > 0.05; cRORA: drusen: *n* = 13, pachychoroid: *n* = 66, mix: *n* = 14, others: *n* = 27, *P* < 0.05). Each error bar indicates standard error

**Fig. 4:**
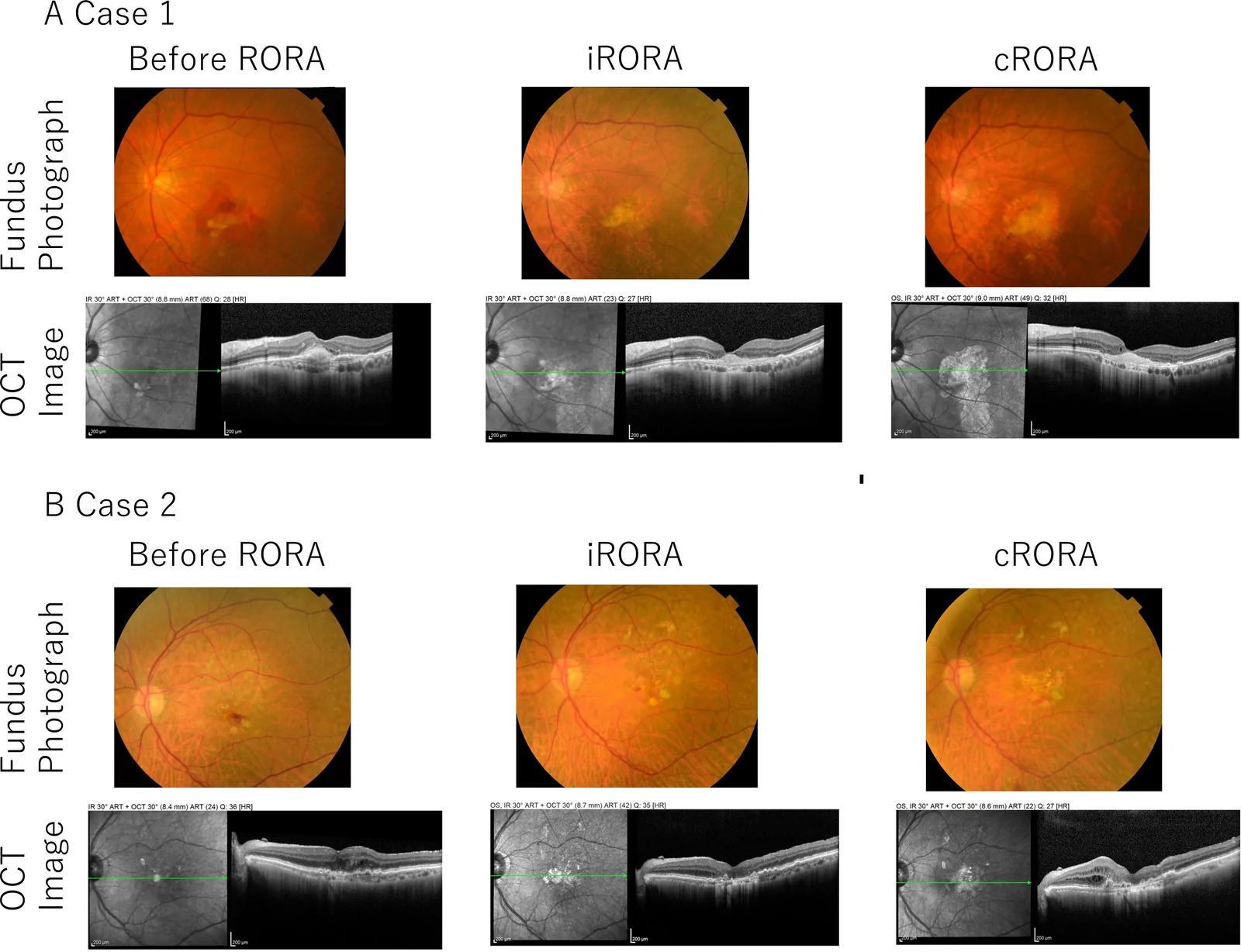
Representative drusen-type cases before the development of retinal pigment epithelial and outer retinal atrophy (RORA), and after the development of incomplete RORA (iRORA) and complete RORA (cRORA). **(A)** and **(B)** Two representative eyes of the drusen type are shown with fundus photographs and optical coherence tomography (OCT) images before any RORA was detectable, at the stage of iRORA, and at the stage of cRORA

We calculated the RORA and cRORA prevalence for each type of nAMD by years (Fig. 3). In the conventional classification, the final prevalence of RORA and cRORA was 44.4/29.6%, 50.0/28.3%, and 83.3/50.0% for tAMD, PCV, RAP, respectively. RAP type showed a trend of higher RORA and cRORA prevalence compared with other type through the examination period, but we did not observe any significant difference between any types in RORA and cRORA prevalence (RORA: *P* > 0.05; cRORA: *P* > 0.05) (Fig. 3A and B). In the novel classification, final prevalence of RORA and cRORA was 76.9/61.5%, 47.0/27.2%, 50.0/14.3%, 40.7/29.6% for drusen type, pachychoroid type, mixed type, others type, respectively. Drusen type had a higher prevalence of RORA and cRORA compared with other types through the examination period. Chi-square test with subsequent post-hoc analysis showed a significantly higher cRORA prevalence but not RORA prevalence in the drusen type compared to other types at the final year of the examination (RORA: *P* > 0.05; cRORA: *P* < 0.05) (Fig. 3C and D). In Fig. 4, we showed two representative cases of drusen type along with their fundus photographs and OCT images developing incomplete RORA (iRORA) and cRORA (Fig. 4A and B).

### Emergence of RORA and cRORA for Different Types of nAMD under Anti-VEGF Therapy

**Fig. 5:**
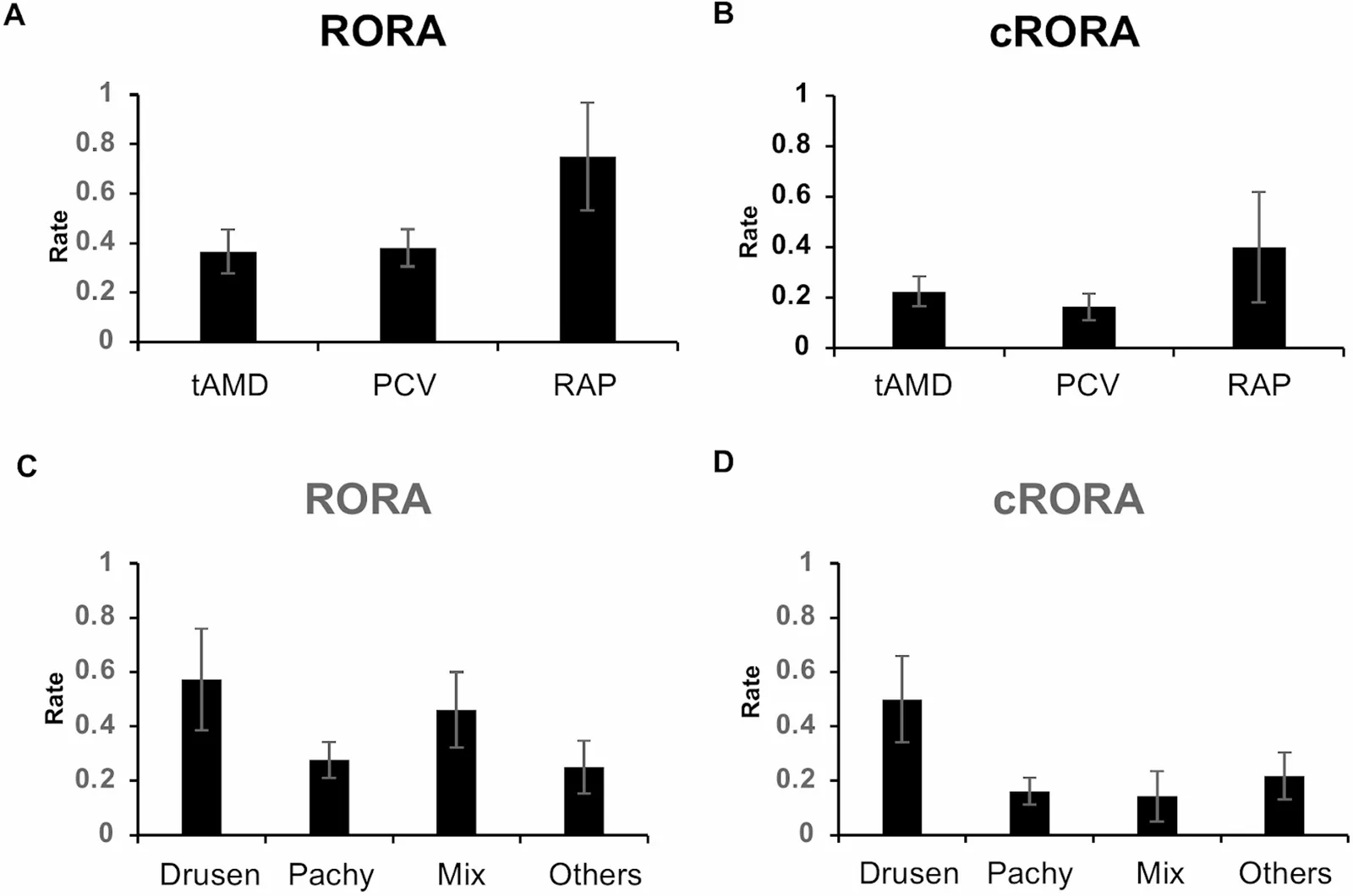
The emergence of the retinal pigment epithelium and outer retinal atrophy (RORA) and complete RORA (cRORA) for different types of neovascular age-related macular degeneration (nAMD). **(A)** Emergence rate of RORA in nAMD patients classified by the conventional classification; **(B)** Emergence rate of cRORA. The chi-square test showed no differences in the emergence rates of both RORA and cRORA among types (RORA: typical AMD (tAMD): *n* = 30, polypoidal choroidal vasculopathy (PCV): *n* = 42, retinal angiomatous proliferation (RAP): *n* = 4, *P* > 0.05; cRORA: tAMD: *n* = 49, PCV: *n* = 49, RAP: *n* = 5, *P* > 0.05). **(C)** Classifying the nAMD patients by the novel classification, we evaluated the emergence rate of RORA; **(D)** of cRORA. The chi-square test showed no difference in both RORA and cRORA emergence rate between types (RORA: drusen: *n* = 7, pachychoroid: *n* = 47, mix: *n* = 13, others: *n* = 20, *P* > 0.05; cRORA: drusen: *n* = 10, pachychoroid: *n* = 56, mix: *n* = 14, others: *n* = 23, *P* > 0.05). Each error bar indicates standard error

We evaluated the new emergence of RORA and cRORA for each type of nAMD under anti-VEGF therapy (Fig. 5). In the conventional classification, RAP type showed a trend of higher prevalence of new RORA and cRORA emergence compared with other types, but chi-square test found no difference among types in new RORA and cRORA emergence rate (RORA: tAMD: *n* = 30, PCV: *n* = 42, RAP: *n* = 4, *P* > 0.05; cRORA: tAMD: *n* = 49, PCV: *n* = 49, RAP: *n* = 5, *P* > 0.05) (Fig. 5A and B). In the novel classification, mixed type and drusen type showed a trend of higher prevalence of new RORA emergence compared with other types, but the chi-square test suggested no significant difference among types in new RORA emergence rate (drusen: *n* = 7, pachychoroid: *n* = 47, mix: *n* = 13, others: *n* = 20, *P* > 0.05) (Fig. 5C). We also observe a trend of higher prevalence of new cRORA emergence in drusen type compared with other types by the novel classification, but chi-square test indicated no difference among types in new cRORA emergence rate (drusen: *n* = 10, pachychoroid: *n* = 56, mix: *n* = 14, others: *n* = 23, *P* > 0.05) (Fig. 5D).

## Discussion

In this study, we classified nAMD patients using either the conventional classification or the novel classification proposed by Yanagi [9]. We investigated the long-term BCVA retention of each type under anti-VEGF therapy. This is the first report demonstrating a significantly lower retention rate in the drusen type and a significantly higher retention rate in the pachychoroid type compared to other types using the novel classification. In contrast, no difference in retention rate was observed between types using the conventional classification. We demonstrated that the emergence of RORA and cRORA was associated with the deterioration of BCVA and that the drusen type of the novel classification showed a higher prevalence of RORA and cRORA.

In the current study, BCVA worsened before and after the emergence of RORA. RORA represented a disorder of photoreceptor and RPE cells [11]. that were directly related to BCVA. A previous study has indicated that the emergence of RORA was accompanied by degeneration of photoreceptors in the retina, leading to the deterioration of BCVA [14, 20]. RORA is also characterized by the loss of RPE cells, which are essential for photoreceptor nutrition and waste removal [21]. Loss of RPE function significantly impacted the maintenance of photoreceptors, resulting in decreased BCVA [21].

In this study, we showed that higher prevalence of cRORA in the drusen type compared to the other types of nAMD classified by the novel classification. The lower incidence of cRORA and better visual preservation observed in the pachychoroid-type subgroup may reflect differences in the underlying pathophysiology compared with drusen-driven neovascular AMD. Pachychoroid spectrum diseases are characterized by choroidal thickening, pachyvessels, and choroidal vascular hyperpermeability, with relatively less accumulation of drusen and complement-mediated retinal pigment epithelium damage [22]. Drusen, lipid-rich deposits that accumulate under the RPE, is a hallmark of AMD [23]. Drusen ooze was associated with a 20-fold higher risk of progression to incomplete (i) RORA and cRORA in a multicenter retrospective study [24]. Drusen collapse has been regarded as the factor most correlated with the risk of progression to iRORA and cRORA [25]. Our recent study has shown that subretinal drusenoid deposits and drusen were associated with more than 6-fold and 4-fold higher risk of RORA progression, respectively [26]. Holz et al. found that a chronic inflammatory response was induced by drusen in the RPE, which ultimately resulted in the degeneration of RPE and photoreceptor cells [27]. This chronic inflammatory response might be associated with the progression of cRORA. A previous study has suggested that activation of the complement system was also promoted by drusen inducing the RPE and photoreceptor cells damage [28]. Complement activation might have a direct impact on the progression of cRORA. Further, it has been reported that oxidative stress and inflammation were important in the pathogenesis of AMD [29]. Drusen formation was associated with oxidative stress [30], which caused RPE dysfunction [31]. As RPE played a crucial role in photoreceptor maintenance [32], its dysfunction promoted photoreceptor degeneration. The effects of oxidative stress on RPE and photoreceptors might contribute to the development of cRORA. Curcio et al. indicated that large drusen interfere with metabolic exchange between the RPE and photoreceptors, leading to cell death [33]. In summary, the higher rate of cRORA prevalence in the drusen type might be attributed to chronic inflammation, complement system activation, oxidative stress, and RPE dysfunction. These mechanisms promote RPE and photoreceptor degeneration and contribute to the development of cRORA. Consequently, the mechanisms leading to retinal atrophy may be less prominent in pachychoroid-associated neovascularization, potentially contributing to a lower risk of cRORA development and better preservation of retinal structure and visual function [34, 35]. These findings suggest that the risk and progression of retinal atrophy may differ according to the underlying disease phenotype and highlight the importance of considering pachychoroid pathophysiology when optimizing long-term management strategies for Asian patients with neovascular AMD [36]. Our findings suggest that the novel classification system may provide a more comprehensive understanding of disease progression and treatment response, potentially guiding more personalized and effective management strategies for patients with nAMD.

All patients in the present study were treated according to the PRN regimen, which generally results in fewer injections and lower cumulative anti-VEGF exposure compared with the treat-and-extend regimen [37]. Although repeated anti-VEGF therapy has been proposed as a potential risk factor for MA, current evidence does not establish a causal relationship between anti-VEGF exposure and MA development [38, 39]. Conversely, Sadda et al. regarded treating exudative MNV adequately as the best option to optimize vision outcomes in nAMD patients [38]. Furthermore, a recent review has indicated that the potential risk of MA associated with anti-VEGF therapy is outweighed by the risk of vision loss resulting from undertreatment [39]. Other studies have also reported that PRN regimens tend to result in undertreatment in clinical practice, and treatment intensity should not be reduced in concern to the theoretical risk of MA [40]. We suggested that disease activity should be adequately controlled while balancing the potential risk of atrophy. Future studies including subgroup specific treatment-burden analysis are needed to determine whether treatment regimens can be optimized to achieve an appropriate balance between exudation control and MA risk by specific type of nAMD. Furthermore, early identification of patients at high risk for MA may facilitate the development of personalized therapeutic approaches.

There were some limitations in the current study. First, the subgroup sample sizes, especially RAP, were small, so the reliability of subgroup comparisons was limited and exploratory. Second, selection bias may have been present since participants were recruited solely from Keio University Hospital. Third, different anti-VEGF agents (Pegaptanib, Ranibizumab, or Aflibercept) were used, which could introduce inconsistencies among participants. Fourth, for patients in whom anti-VEGF treatment was administered to both eyes during the study period, only the first-treated eye was included to avoid inter-eye correlation in this single-eye-per-patient analysis. Future studies should consider bilateral-eye analyses using mixed-effects models or generalized estimating equations. Finally, we could not establish causality as it was a retrospective study, and we should perform a prospective study in the future.

## Conclusions

We found a lower BCVA retention rate in the drusen-type disease, which might be related to higher cRORA prevalence, as cRORA is associated with BCVA decline. Because this was a retrospective observational study, causality cannot be established, and prospective studies are warranted. The novel classification may better capture long-term visual prognosis and atrophy burden, potentially guiding more individualized management of nAMD.

## Data Availability

DH and SK had full access to all of the study data and take responsibility for the integrity of the data and the accuracy of the data analysis. The datasets generated and/or analyzed during the current study are not publicly available because they were used under institutional license, but are available from the corresponding author on reasonable request and with the permission of YT.

## Abbreviations

nAMD: Neovascular age-related macular degeneration
CNV: Choroidal neovascularization
anti-VEGF: Anti-vascular endothelial growth factor
tAMD: typical nAMD
PCV: Polypoidal choroidal vasculopathy
RAP: Retinal angiomatous proliferation
MNV: Macular neovascularization
RPE: Retinal pigment epithelium
MA: Macular atrophy
CAM: Classification of atrophy meetings
RORA: Retinal pigment epithelial and outer retinal atrophy
cRORA: Complete retinal pigment epithelial and outer retinal atrophy
PRN: Pro re nata
PDT: Photodynamic therapy
FA: Fluorescein angiography
ICGA: Indocyanine green angiography
VA: Visual acuity
BCVA: Best-corrected visual acuity
SD-OCT: Spectral-domain optical coherence tomography
IRF: Intraretinal fluid
SRF: Subretinal fluid
sub-RPE fluid: Subretinal pigment epithelial fluid
